# Integrated respiratory and palliative care leads to high levels of satisfaction: a survey of patients and carers

**DOI:** 10.1186/s12904-019-0390-0

**Published:** 2019-01-19

**Authors:** Natasha Smallwood, Thomas Moran, Michelle Thompson, Peter Eastman, Brian Le, Jennifer Philip

**Affiliations:** 1 Department of Respiratory and Sleep Medicine, The Royal Melbourne Hospital, Royal Parade, Parkville, Victoria 3050 Australia; 2 Department of Medicine (Royal Melbourne Hospital), University of Melbourne, Parkville, Victoria 3050 Australia; 3 The Melbourne Medical School, University of Melbourne, Parkville, Victoria 3050 Australia; 4 Department of Palliative Care, The Royal Melbourne Hospital, Parkville, Victoria 3050 Australia; 5 Chair of Palliative Medicine, University of Melbourne, St Vincent’s Hospital and Victorian Comprehensive Cancer Centre, Melbourne, Australia; 6 Department of Palliative Care St Vincent’s Hospital, Victoria Parade, Fitzroy, Victoria 3065 Australia

**Keywords:** Attitudes, Survey, Satisfaction, Palliative care, Integrated care, Dyspnea

## Abstract

**Background:**

The Advanced Lung Disease Service is a unique, new model of integrated respiratory and palliative care, which aims to address the unmet needs of patients with advanced, non-malignant, respiratory diseases. This study aimed to explore patients’ and carers’ experiences of integrated palliative care and identify valued aspects of care.

**Methods:**

All current patients of the integrated service and their carers were invited to complete a confidential questionnaire by post or with an independent researcher.

**Results:**

Eighty-eight responses were received from 64 (80.0%) eligible patients and from 24 (60%) eligible carers. Most participants (84, 95.5%) believed the integrated service helped them to manage breathlessness and nearly all participants (87, 98.9%) reported increased confidence managing symptoms. One third of patients (34.4%) had received a nurse-led domiciliary visit, with nearly all regarding this as helpful.

Most participants believed the integrated respiratory and palliative care team listened to them carefully (87, 98.9%) with opportunities to express their views (88, 100%). Highly valued aspects of the service were continuity of care (82, 93.2%) and long-term care (77, 87.5%). Three quarters of participants (66, 75.0%) rated their care as excellent, with 20.5% rating it as very good. Nearly all (87, 98.9%) participants reported that they would recommend the service to others.

**Conclusions:**

Patients and carers expressed high levels of satisfaction with this model of integrated respiratory and palliative care. Continuity of care, high quality communication and feeling cared for were greatly valued and highlight simple but important aspects of care for all patients.

**Electronic supplementary material:**

The online version of this article (10.1186/s12904-019-0390-0) contains supplementary material, which is available to authorized users.

## Background

Palliative care aims to improve the quality of life of patients and their families when facing life-threatening illness, through the prevention and relief of suffering [1]. While specialist palliative care originally arose in response to the end-of-life needs of patients with cancer, it’s goals of providing holistic care to manage distressing symptoms, psychosocial and spiritual issues, are equally relevant to patients with advanced, non-malignant diseases. Therefore many guidelines recommend palliative care for patients with advanced, non-malignant respiratory disease [2–6]. However, few patients with end-stage chronic obstructive pulmonary disease (COPD) access specialist palliative care [7]. In the United Kingdom (UK) and Australia only 16.7–17.9% of patients with COPD access any specialist palliative care [8–10].

Given the unmet palliative care needs of patients with advanced, non-malignant, respiratory disease [7, 9, 11, 12], new, accessible services are required, which offer individualised, integrated, palliative care together with disease-directed, respiratory care. Ideally such services should build on existing services provided by respiratory medicine or rehabilitation teams [13, 14] and should aim to reduce patients’ fears of abandonment by their treating team and offer extended consultations so that there is sufficient time to discuss complex issues [15].

The Advanced Lung Disease Service (ALDS) in Australia is one such model and has been shown to be associated with improved outcomes including enhanced active management of breathlessness, increased advance care planning, greater access to palliative and end-of-life care and reduced unscheduled healthcare utilisation [16]. Patients’ experiences and satisfaction are central in the assessment of healthcare quality, however, little is known regarding patients’ or carers’ attitudes to new models of integrated respiratory and palliative care. This study aimed to assess patients’ and carers’ experiences of the ALDS, identify valued aspects of the service and determine priorities for service development.

## Methods

### Study setting

The Advanced Lung Disease Service (ALDS) is a multidisciplinary, single point-of-access, integrated respiratory and palliative care service, based within a major Australian teaching hospital. The service is a partnership between respiratory and palliative medicine and focuses on active symptom management, individualised patient and carer education (including providing written resources), and advance care planning. The ALDS accepts all referrals for patients with severe, non-malignant, respiratory disease, with no set referral criteria. Either long-term care (over the last few years of life) shared with the primary care team or short-term care are offered [16]. Patients and any accompanying carers are usually seen together (unless they request to be seen separately) in the ALDS clinic, where all patients meet a respiratory physician and nurse specialist who both have expertise in palliative care, and the majority also meet a palliative care doctor. Home visits from the ALDS respiratory nurse specialist are also offered according to patients’ needs. Additionally, the ALDS provides support to manage psychological issues from a psychologist in the ALDS clinic, a nurse-led telephone support service, a regular multidisciplinary team meeting and case conferences with community health teams and primary care [16].

### Survey design

From August 2016 to February 2017 all current ALDS patients were invited to complete a voluntary, confidential survey questionnaire (Additional file 1: Appendix S1) regarding their experiences of and satisfaction with the ALDS. Previously discharged patients were excluded, principally to avoid causing distress to relatives if the patient had died since discharge, and secondly to avoid limited recall of experiences if they had not seen the ALDS for some time. Current patients’ carers were invited to participate if they had had ever attended the ALDS clinic with their relative or been present during an ALDS home visit.

The questionnaire was modelled on the patient satisfaction assessment tool used by Reilly et al [17], which explored patients’ attitudes to the London Breathlessness Support Service, a similar model of integrated respiratory and palliative care. Survey questions focussed on four main themes:

1. ALDS hospital clinic - including usefulness of clinic visits, symptom management, health information discussions and waiting time to be seen
2. ALDS nursing support service - including types of telephone support accessed and home visits
3. General views and overall opinion - including confidence in the service, feeling heard and respected, having enough time and opportunities to discuss important aspects of care, and valued elements of the service
4. Areas for future improvement.

### Survey distribution

Patients and carers were given the choice of completing the survey questionnaire by post, or at a hospital outpatient appointment where they could either complete the paper questionnaire independently, or as a face-to-face structured interview with a researcher (TM) who was independent of the ALDS clinical team. Patients and carers who completed the survey by interview, were spoken to separately where possible. Paper copies of the questionnaire (as well as a separate letter explaining the options for completing the survey) were posted to all current ALDS patients, then if not returned, four weeks later each patient was telephoned by the independent researcher to remind them of the different options for survey completion.

Postal survey questionnaires were each assigned a unique participant research code in order to track survey completion and to link patient survey responses with demographic data. Only the independent researcher interviewed participants, accessed individual survey questionnaires and knew which participants completed the study, thus ensuring patients’ opinions could not affect or be perceived to affect future clinical care. Informed consent was either sought verbally for patients completing the survey at a face-to-face interview, or implied for patients returning the survey questionnaire by post. Ethics approval was granted by Melbourne Health (Approval number: QA2015077).

### Data analysis

Data are reported descriptively using counts and frequencies. Patients’ and carers’ responses, and ALDS patient respondents’ and non-respondents’ demographic data were compared using the Pearson Chi-Square test (for proportions) or Student’s t test (for continuous numerical data). Statistical analyses were performed using IBM SPSS Statistics (Chicago, IL) version 24, with a *p*-value of less than 0.05 indicating statistical significance. Free text comments were extracted and transcribed separately. TM analysed the comments using a descriptive and exploratory thematic analysis framework to identify themes until thematic saturation was reached. Both the free text comments and themes were reviewed (by NS and TM) and following discussion, refinement and consensus the final themes were agreed.

## Results

One hundred and fifty-five patients accessed the ALDS before commencement of the survey, of whom 54 patients had died and 15 had been discharged. Of the 86 current patients, 6 did not speak English and were therefore excluded. Eighty-eight participants completed the survey, including 64 (80.0%) patients and 24 (60.0%) responses from 40 eligible carers. Forty-two surveys were completed by patients, without receiving responses from any eligible carers, and 2 responses were received from carers without receiving a completed questionnaire from the patient. Twenty-two patient and carer dyads participated (i.e. individual responses were received from both the patient and their carer). Of the participants, thirty-nine (60.9%) patients and 11 (45.8%) carers completed the questionnaire as individual, face-to-face interviews in clinic and 25 (39.1%) patients and 13 (54.2%) carers returned the survey by post. Patients had attended a median of eight (IQR = 4–12) ALDS appointments over a median time period of 21.3 (IQR = 8.4–36.9) months. Carer participants had a median of 5 (IQR = 3–12) episodes of contact with the ALDS team. There was no significant difference between survey respondents’ and non-respondents’ demographic characteristics (Table 1) or between carers’ and patients’ responses for any survey question.

**Table 1:** Participants’ characteristics

	Eligible Patients (*n* = 80)	Respondents (*n* = 64)	Non-respondents (*n* = 16)
Age	75 (71–81)	75 (71–80)	77 (74–84)
Male	42 (52.5%)	34 (53.1%)	8 (50%)
Lives alone	31 (38.8%)	25 (39%)	6 (37.5%)
COPD	75 (93.8%)	60 (93.8%)	16 (100%)
Bronchiectasis	4 (5.0%)	4 (6.2%)	0
Pulmonary Fibrosis	1 (1.2%)	0	0
Anxiety and/or Depression	38 (47.5%)	29 (45.3%)	9 (56.3%)
FEV_1_% predicted	40 (31–50)	41 (30–50)	40 (32–50)
Forced expiratory ratio (%)	34 (29–44)	33 (28–45)	35 (30–41)
DLco	8 (7–10)	9 (7–11)	7 (7–9)
MMRC Dyspnoea Score	3 (2–4)	3 (2–4)	3 (2–4)
Home Oxygen Use	45 (56.3%)	36 (56.3%)	9 (56.3%)
Past completion of pulmonary rehabilitation program	71 (80.7%)	58 (90.6%)	13 (81.3%)
Number of ALDS appointments	7.5 (4–11)	8 (4–12)	6 (4–9)
Accessed specialist palliative care in ALDS clinic	66 (82.5%)	52 (81.3%)	14 (87.5%)

Data are represented as counts or medians with frequencies or interquartile ranges respectively in parentheses. *FEV1* Forced Expiratory Volume in 1 s. *FVC* Forced Vital Capacity. *DL*_*CO*_ Diffusion capacity of the lung for Carbon Monoxide. *mMRC* Modified Medical Research Council Dyspnoea Score

### ALDS clinic management

Seventy-seven (87.5%) participants (56 patients and 21 carers) reported that their ALDS clinic visits were definitely helpful, with the remainder reporting they were somewhat helpful. Participants reported (in free text comments) that clinic visits were considered helpful because they valued: explanations and advice related to symptoms and the underlying condition (35, 39.8%), optimal disease management (24, 27.3%), respiratory disease monitoring (14, 15.9%), advice on coping strategies (16, 18.2%) and the approachable and caring nature of the ALDS team (23, 26.1%) (Table 2).

**Table 2:** Participants’ perceptions of why the ALDS clinic is helpful

Theme 1: Explanations and advice related to symptoms and the condition	
*“…(the respiratory specialist) and …(the respiratory nurse) explain everything for you, so you’re not left in the dark. They take the time to let you know what’s going on.”* *“The assessment and review of my current condition is most helpful to me in monitoring the condition. Together with the explanation of what is happening and why it is happening assists me to gain some control over the condition.”* *“When I’m worried about something, if I have a chat with …(the respiratory specialist) and …(the respiratory nurse), it settles me. It really helps to talk about everything.”*	
Theme 2: Optimal management and monitoring of the condition	
*“They take me through everything, and get all my tests.”* *“You feel that it’s all been checked, and you feel confident in yourself. There is nothing to worry about because you feel safe in their hands.”* *“It’s great to have access to a service that gives me confidence that I am receiving the best available and up to date assistance for my condition, in a very friendly manner.”.*	
Theme 3: Kind and caring ALDS team	
*“They are very attentive and go out of their way to look after me. It feels very personal and I’m treated with respect. It shows they really care and that I’m not just a number. I’m very happy with the service I am receiving. I couldn’t ask for any better.”* *“I look forward to it and listen to what they’re saying. They take the time and let you know what’s going on”.* *“I am treated very well. The kindness is appreciated”*	

Examples of some of the illustrative quotes provided by participants are included

Almost two thirds (57, 64.8%) of participants reported not waiting long to be seen in the ALDS clinic, with a further 17 (19.3%) and 13 (14.8%) reporting wait times were less than or similar to other hospital clinics respectively. All carers (100%) and nearly all patients (62, 96.9%) reported being given enough time to discuss the underlying condition and treatment.

The majority of participants believed the ALDS team had helped them to manage symptoms and nearly all participants (87, 98.9%) reported having increased confidence to manage symptoms after seeing the ALDS team (Table 3). Sixty (93.8%) patients reported receiving help managing breathlessness, with 59 (92.2%) patients documented by the medical team as having severe breathlessness (mMRC = 3–4) and 5 (7.8%) having moderate breathlessness (mMRC = 2). Similarly, twenty-five (39.1%) patients reported receiving help with mood problems, with 29 (45.3%) known to have a medically confirmed diagnosis of anxiety and/or depression.

**Table 3:** ALDS symptom support

The ALDS has tried to help with the following symptoms		
	Patients: ‘Yes’	Carers: ‘Yes’
Breathlessness	60 (93.8%)	24 (100%)
Cough	43 (67.2%)	16 (66.7%)
Mood problems (anxiety or depression)	25 (39.1%)	10 (41.7%)
Poor appetite or low weight	24 (37.5%)	9 (37.5%)
Sleeping problems	25 (39.1%)	12 (50%)
Constipation	13 (20.3%)	9 (37.5%)
Nausea or vomiting	11 (17.2%)	5 (20.8%)
Since seeing the ALDS team, do you feel more confident self-managing these symptoms?		
Yes, definitely	49 (76.6%)	18 (75%)
Yes, somewhat	14 (21.9%)	6 (25%)
No	1 (1.6%)	0

**Fig. 1:**
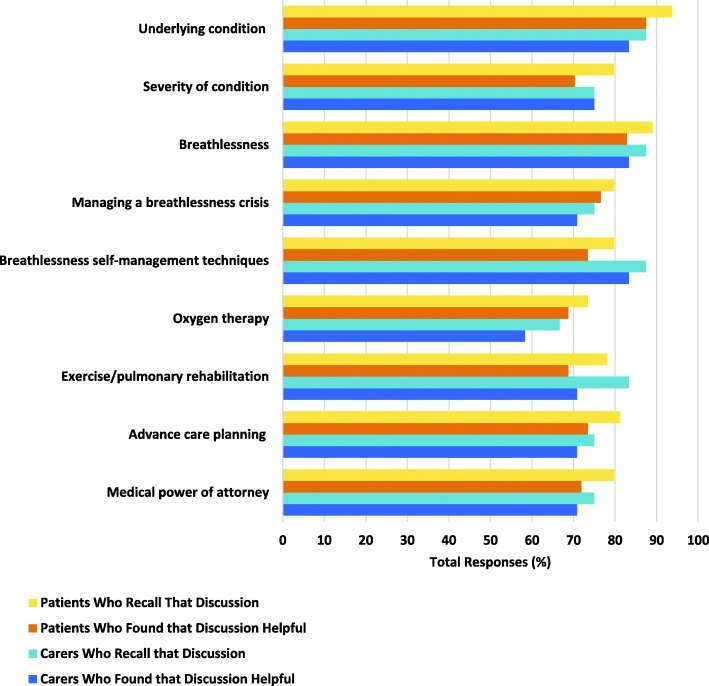
Topics discussed with ALDS patients and carers

Patients and carers could recall discussing multiple aspects of their illness and care with the ALDS team and found these conversations helpful (Fig. 1). Three quarters (48, 75%) of patients and 66.7% of carers did not want further written information in addition to discussing these topics, with only 8 (12.5%) patients and 7 (29.2%) carers requesting these resources.

Nearly all participants (84, 95.5%) reported being given the opportunity to discuss topics they wanted to raise with the ALDS team. Topics participants reported discussing (in free text comments) included: the current condition and changes in disease state (20, 25%), medications (14, 17.5%), symptoms (11, 13.8%), and other available treatments (8, 10%). Additionally, 13 (16.3%) participants reported being able to discuss anything with the ALDS team and 7 (8.8%) discussed other, non-respiratory, medical conditions with the team.

### ALDS respiratory nursing service

In addition to seeing the specialist respiratory nurse in the ALDS clinic, approximately half of patient (31, 48.4%) and carer (12, 50.0%) participants recalled accessing the ALDS nurse-led telephone service. All aspects of the telephone support service were considered helpful by patients and carers who accessed them. One third of patient (34.4%) and carer (33.3%) participants recalled receiving a home visit from the ALDS specialist respiratory nurse, with most participants (95.6% of patients and 87.8% of carers) who accessed this service regarding this as helpful.

### ALDS care generally

The majority of participants believed that the ALDS team listened to them carefully (87, 98.9%) and gave them opportunities to express their views (88, 100%)(Table 4). The most highly valued aspects of the service were continuity of care (82, 93.2%) and long-term care (77, 87.5%)(Table 5). Overall, three quarters of participants (66, 75.0%) rated their care from the ALDS as excellent, with 20.5% rating it as very good. Nearly all (87, 98.9%) participants reported that they would recommend the ALDS to others.

**Table 4:** Participants’ beliefs regarding ALDS care

Does the ALDS team listen carefully to you?		
	Patients	Carers
Yes, definitely	59 (92.2%)	21 (87.5%)
Yes, somewhat	5 (7.8%)	2 (8.3%)
No	0	0
Don’t know	0	1 (4.2%)
Are you given a chance to express your views with the ALDS team?		
Yes, definitely	59 (92.2%)	22 (91.7%)
Yes, somewhat	5 (7.8%)	2 (8.3%)
No	0	0
Don’t know	0	0
Do you have enough say in decisions about treatment or care?		
Yes, definitely	55 (85.9%)	21 (87.5%)
Yes, somewhat	7 (10.9%)	3 (12.5%)
No	0 (0.0%)	0 (0.0%)
Don’t know	2 (3.1%)	0 (0.0%)
Does the ALDS team treat you with respect and dignity?		
Yes, definitely	60 (93.8%)	23 (95.8%)
Yes, somewhat	2 (3.1%)	1 (4.2%)
No	0	0
Don’t know	2 (3.1%)	0
Do you have trust and confidence in the ALDS team?		
Yes, definitely	57 (89%)	23 (95.8%)
Yes, somewhat	6 (9.4%)	1 (4.2%)
No	1 (1.6%)	0
Don’t know	0	0

**Table 5:** Valued aspects of ALDS care

What aspects of our service are important to you or your relative?		
	Patients	Carers
Continuity of care	59 (92.2%)	23 (95.8)
Long term care	56 (87.5%)	21 (87.5%)
Urgent Review	47 (73.4%)	16 (66.7%)
Nurse specialist telephone support	43 (67.2%)	11 (45.8%)
Nurse specialist visits the home	31 (48.4%)	10 (41.7%)
Extended consultations	36 (56.3%)	13 (54.2%)
Afternoon appointments	25 (39.1%)	9 (37.5%)
Palliative and supportive care doctor	25 (39.1%)	10 (41.7%)
In the ALDS clinic would you like to see any other health professionals?^a^		
No	49 (76.6%)	20 (83.3%)
Physiotherapist or Occupational therapist	16 (25%)	6 (37.5)
Psychologist	2 (3.1%)	1 (4.17%)
Palliative and supportive care nurse	3 (4.7%)	0

^a^Multiple response options were possible for this question

### Service development

The majority of patients (49, 76.6%) and carers (20, 83.3%) did not wish to see any additional health professionals during their ALDS clinic visits (Table 5). Of sixty-four responses received regarding whether there was any other help or support participants wanted to receive from the ALDS team, or if the service could do anything better, 62 participants answered “no”. One participant suggested shorter waiting times in the clinic and one participant suggested the clinic should run multiple times each week so they there was greater flexibility regarding which day to attend. Themes highlighted in free text comments regarding the service generally were of: gratitude to the service (11, 12.5%), receiving excellent care (30, (34.1%) and a kind, caring service (7, 8.0%).

## Discussion

In this study, which had a very high response rate and is the first structured survey to include carers receiving support from an integrated respiratory and palliative care service, participants reported a very, high level of satisfaction with three quarters rating their care as excellent, nearly all recommending the ALDS to others and all participants finding the ALDS helpful. Patients and carers highly valued and gained increased confidence from long-term specialist management and opportunities to discuss multiple aspects of their healthcare with a trusted, expert and knowledgeable team, which spent time listening to them. Additionally, acknowledgement and validation of symptom burden, reassurance with explanations and provision of coping strategies, and feeling supported by an attentive team, were also highly appreciated. These responses highlight the profound importance of creating a therapeutic relationship, offering individualised care, which respects each person’s autonomy and high quality communication, for all health professionals not just those working in palliative care.

Respiratory patients managed by two holistic breathlessness services (the London Breathlessness Support Service (UK) and Cambridge Breathlessness Intervention Service (UK)), as well as patients managed by the Canadian integrated respiratory and palliative care service “INSPIRED” have also reported high levels of satisfaction and greatly valued similar aspects of care [17–19]. However, importantly this study is the first to specifically examine which components of integrated care participants found helpful. The willingness of both patients and their carers to accept integrated respiratory and palliative care and their high levels of satisfaction from such care, highlight the limited access they currently have to traditional models of palliative care [7, 9, 11, 12]. Notably, while there is not one “right” model of integrated palliative care, patients managed by the Cambridge Breathlessness Intervention Service (which offers short term support) also reported valuing contact with the team after their scheduled visits were completed [18]. However, while longer term interventions, which offer continuity of care, may be preferred by patients and their carers, integrated respiratory and palliative care services must be accessible and sustainable. Therefore close working relationships with primary care and other community health teams, together with flexibility to accept referrals promptly in response to individuals’ changing health are essential.

It is well recognised that patients with COPD often have limited understanding of their disease and symptoms [20, 21]. Similarly, patients with advanced COPD have a variable illness trajectory usually punctuated by acute exacerbations [22], which may require admissions and interventions such as non-invasive ventilation that can be frightening and challenging for patients [23]. Additionally, many patients with COPD have co-existing psychological issues [24, 25] and multiple medical comorbidities (any of which may impair their ability to retain information) that they must manage simultaneously each day. Therefore, these patients have significant ongoing health information needs [20]. In this study, despite the advanced illness stage and the fact that nearly all patients had previously completed pulmonary rehabilitation (in which education is a core component), both patients and carers highly valued information regarding the underlying illness and self-management education. This finding highlights the benefit of ongoing education for patients and carers that is relevant to their experiences, and builds on previous discussions to provide information to address symptoms, disease state, fears and existential issues.

COPD patients see themselves as living with, not dying from, their COPD [21]. Similarly, patients may fear abandonment by their usual treating team as they approach the end of life [15], therefore the involvement of respiratory clinicians, who are seen as actively treating the underlying condition, is essential within an integrated palliative care team. In our study, and also in the survey of patients cared for by the London Breathlessness Support Service [26], patients highly valued respiratory medicine involvement and disease monitoring. By contrast while the majority of patients saw a specialist palliative care doctor in the ALDS clinic, this was considered important by less than half of patients. However, the ALDS respiratory team has completed additional training in palliative care. Therefore, supported in the clinic by specialist palliative care doctors, the respiratory team delivers many elements of palliative care, including prescribing and managing opioids for refractory breathlessness. Consequently, while patients may not perceive the benefit of palliative care physicians, these clinicians are essential in supporting the provision of palliative care by the respiratory team. Equally increasing the competence of the respiratory team to provide such care facilitates increased access to palliation and palliative care [16].

Supporting patients at home (through a telephone support service and home visits) was also highly valued by patients and carers who accessed these services. Similarly patients cared for by both the London Breathlessness Support Service and the Cambridge Breathlessness Intervention Service also valued home support [17, 18]. Furthermore, though patients with advanced lung disease desire longer consultations to discuss their health, attending medical appointments can be challenging due to breathlessness [27, 28]. Therefore integrated care clinic visits, which enable patients and carers to see two or three health professionals together over an hour, while efficient may be exhausting for patients. Consequently, home visits may overcome patients’ unwillingness to see multiple health professionals during “one stop” clinic visits.

Notably when asked regarding service development opportunities, the participants neither wished to change the composition of the ALDS team, nor did the majority desire any additional services or support from the service. The two recommendations for service improvement were to reduce the waiting time in clinic and to run the ALDS clinic on multiple days of the week, to allow greater choice regarding day to attend. Both suggestions highlight the challenges patients with advanced lung disease and their carers face when trying to attend hospital appointments. Furthermore these recommendations are a timely reminder that the ethos of palliative care (and thus also integrated palliative care) is to provide care that is both patient and family focused, as well as responsive to individual needs [1].

### Limitations of the study

Acquiescence bias (which is the tendency to agree with statements of opinion) may lead to increased levels of consumer satisfaction [29]. However, in this study there was an extraordinarily high patient response rate (80%) and overall a large number of participants, whereas usually consumer satisfaction survey response rates vary from 30 to 60% [17, 30]. The high response rate in our study suggests that the issues addressed within the questionnaire were a priority for patients, there were more satisfied consumers that not, and the questionnaire was easy to understand and relevant to the patient population. Therefore our very high response rate together with the fact that patient participants’ characteristics were representative of the whole ALDS patient cohort, significantly increase the internal validity of these results. Similarly a researcher who was independent of the clinical team undertook patient interviews and de-identified all data for analysis to preserve anonymity and therefore reduce patient concerns that negative feedback may affect their future clinical care. However, neither the questionnaire used in the study nor the one used be Reilly et al (from which this survey questionnaire was developed) have been validated [17].

Surveys require participants to retrospectively recall in detail many aspects of their care that may be forgotten, particularly when receiving long-term care. Collateral data collected from the clinical notes demonstrated that the number of patients who informed the ALDS clinical team that they experienced severe breathlessness and mood disorders, and the number of patients who recalled receiving support for both issues were well matched. Collateral information regarding other symptoms (such as cough, sleep and appetite) would have strengthened this study and identified any gaps in symptom management. While a minority of eligible patients (six) were excluded because they did not speak English, it is unclear how patients from non-English speaking backgrounds view the ALDS.

## Conclusions

Integrated respiratory and palliative care provided by the Advanced Lung Disease Service is associated with very high levels of patient and carer satisfaction. Continuity of care, high quality communication and feeling cared for were greatly valued and highlight simple but important aspects of care. Therefore core components of new integrated respiratory and palliative care services should ideally include: access to palliative care activities (but not necessarily palliative care personnel if the respiratory team can provide this care), health and self-management information and education, and home support. Importantly, multi-site controlled trials are still required to examine on a larger scale the effectiveness (including cost-effectiveness) of integrated palliative care for patients with advanced respiratory disease, as well as further studies to understand patients’ and carers’ perspectives regarding these new models of care.

## Additional file

Additional file 1: Appendix S1. ALDS patient and carer questionnaire. (DOCX 275 kb)

## Abbreviations

ALDS: Advanced Lung Disease Service
COPD: Chronic obstructive pulmonary disease
