# Supplementary material for: Integrated respiratory and palliative care leads to high levels of satisfaction: a survey of patients and carers
Source: BMC Palliat Care. 2019 Jan 19;18:7. doi: 10.1186/s12904-019-0390-0 (PMC6339689; doi:10.1186/s12904-019-0390-0)
Supplement: Supplementary file 1 — Appendix S1. ALDS patient and carer questionnaire. (DOCX 275 kb) [file 12904_2019_390_MOESM1_ESM.docx]

**Appendix S1. Advanced Lung Disease Service patient and carer questionnaire**

**As a patient, or a carer/ relative of a patient, who is looked after by the Advanced Lung Disease Service at The Royal Melbourne Hospital, we are very interested to hear your views about this service. Our service aims to continuously develop and improve, so your views are essential to enable us to provide high quality, patient and family focused care for people with severe lung disease.**

**Please complete this short, voluntary survey which is completely anonymous and confidential. No personal information that could identify you is being collected. If you are completing this survey on your computer your email address will not be linked to your response. Your consent to participate is implied by completing this survey.**

**Please note that if you are a carer, through out the survey we have referred to your relative. We realise that not all carers are related to the person they care for, but we would still value your feedback whatever your relationship is with the person you care for - thank you.**

**The results from this survey are being collected by a researcher (Thomas Moran) who is separate from the clinical team. So the clinical team will not be able to identify individual responses and your clinical care will not be affected in the future.**

**This survey has been approved by the Melbourne Health Research Office. If you have any questions about this survey please contact:**

**Jessica Turner - Manager Melbourne Health Research Ethics Committee (9342 8530) Michelle Thompson - Respiratory Nurse Consultant (0400 367 036)**

**Thank you for completing this survey - your feedback is very important to us.**

# ALDS team


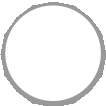

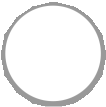


1. Which members of the Advanced Lung Disease Service team have you met at the hospital or spoken to over the telephone? (Please select all that apply)

Dr Natasha Smallwood - Consultant Respiratory Physician Ms Michelle Thompson - Respiratory Nurse Consultant

Dr Peter Eastman - Supportive & Palliative Care Consultant

Supportive & Palliative Care Registrars

1. Are you a patient or a carer/relative of a patient who is looked after by the Advanced Lung Disease Service at The Royal Melbourne Hospital? (Please choose only one response)

Patient

Carer or relative

# Carer ALDS contact


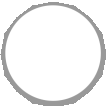

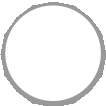


1. As a carer/ relative of a patient, how many times have you had contact with members of the Advanced Lung Disease Service team either face to face in the hospital or by telephone?

Zero

At least once - please specify the number of times

# ALDS hospital clinic


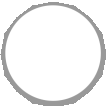

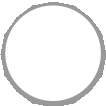

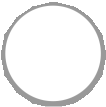

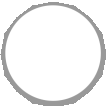


1. Do you find your visits, or the visits of your relative, to the Advanced Lung Disease Service clinic helpful? (Please choose only one)

Yes, definitely helpful Yes, somewhat helpful No, not helpful

Don't know

# ALDS hospital clinic

1. Please tell us why you find your visits, or the visits of your relative, to the Advanced Lung Disease Service clinic ARE helpful

# ALDS hospital clinic

1. Please tell us why you find your visits, or the visits of your relative, to the Advanced Lung Disease Service clinic are NOT helpful

# ALDS hospital clinic


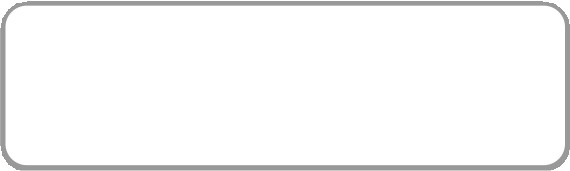

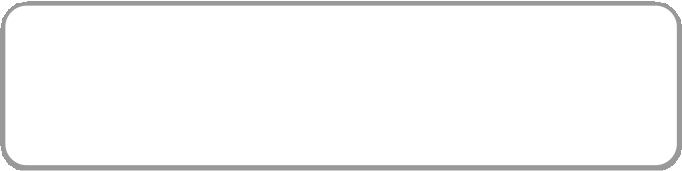

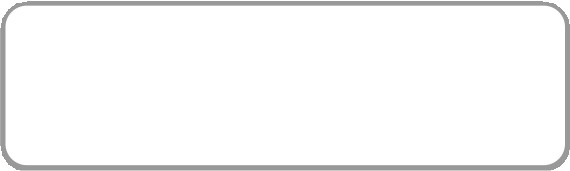

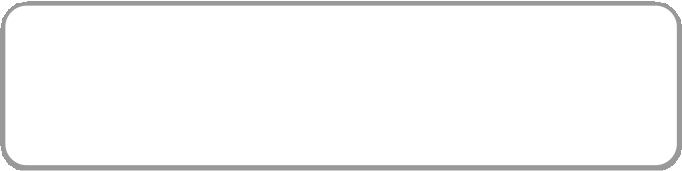

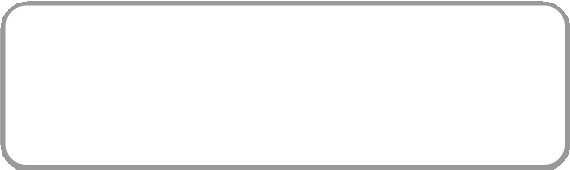

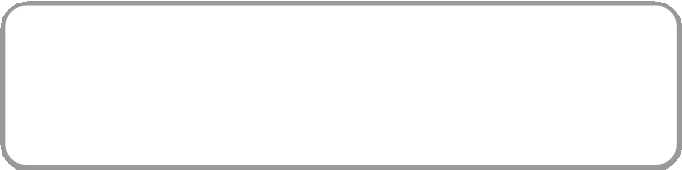

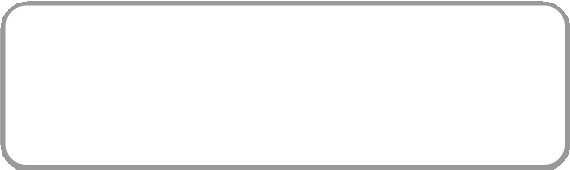

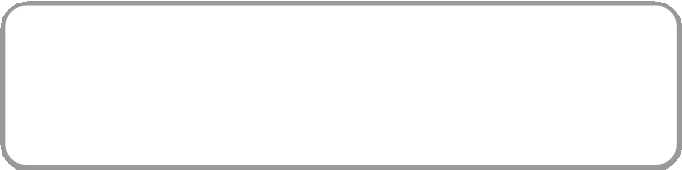

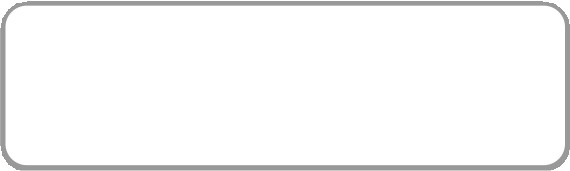

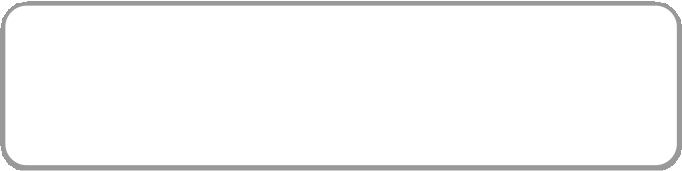

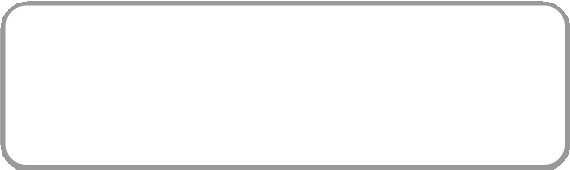

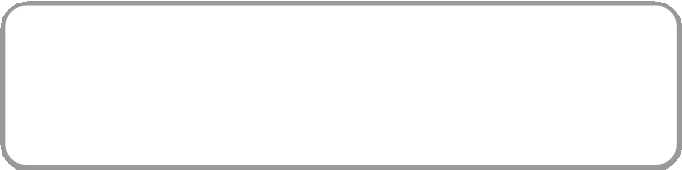

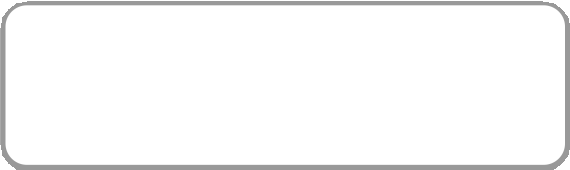

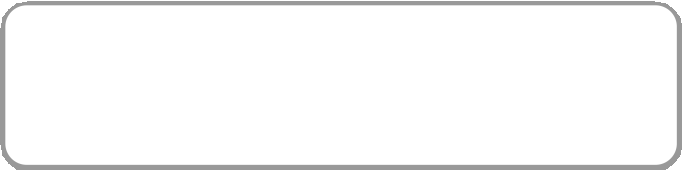

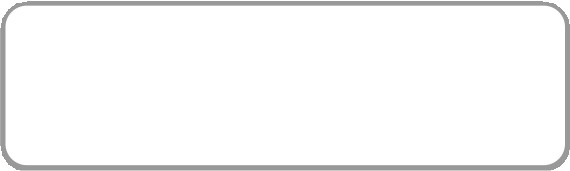

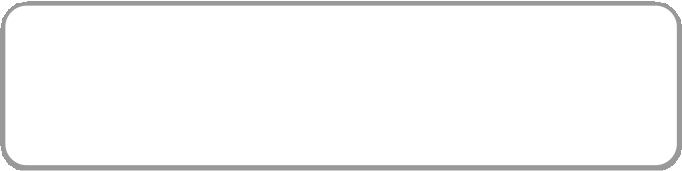

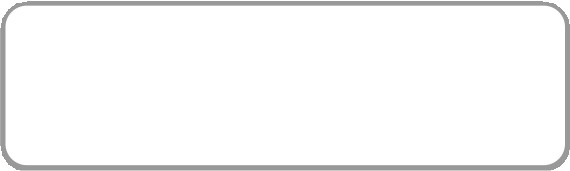

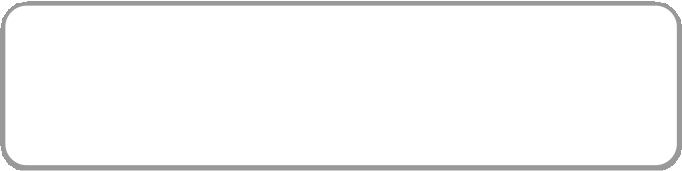


1. During clinic visits, you may have discussed (and been given written information about) many issues related to your health care or your relative's health care. Please tell us if you found these discussions helpful.

Did you discuss this with the team? Was this discussion helpful?

Your (or your relative's) underlying lung condition (diagnosis)

Breathlessness and what causes this

Techniques to self- manage breathlessness (e.g. breathing exercises, using a handheld fan, pacing yourself, relaxation and distraction)

Exercise classes, physiotherapy or community pulmonary rehabilitation classes

Oxygen therapy

How to manage episodes of breathlessness crisis

The severity of your (or your relative's) lung condition and the impact on future health

Advance care planning - the opportunity to tell the team about your (or your relative's) future treatment wishes

Medical power of attorney - who can make decisions for you (or your relative) in the future in case you are unable to do this yourself

# ALDS hospital clinic


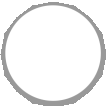

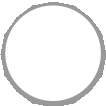

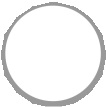

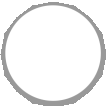

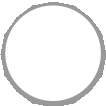

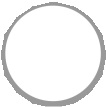

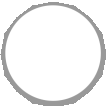

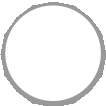


1. Would you like more written information about any of these topics? (Please choose only one)

Don't know No

Yes, I would like written information about (please specify)

1. Has the Advanced Lung Disease Service team tried to help you or your relative with any of the following symptoms? (Please select all that apply)

Breathlessness Cough

Mood problems such as anxiety or depression Poor appetite or low weight

Sleeping problems Constipation Nausea or vomiting

Other (please specify)

1. Since seeing the Advanced Lung Disease Service team, do you feel more confident self-managing these symptoms? (Please choose only one)

Yes, definitely Yes, some what No

Don't know

Other (please specify)

1. Do your Advanced Lung Disease Service clinic visits provide you or your relative with an opportunity to talk about the things you want to discuss? (Please choose only one)


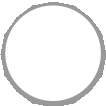
 Yes, definitely


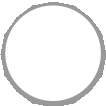

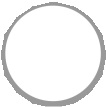
 Yes, to some extent
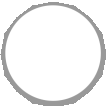
 No

Don't know

# ALDS hospital clinic

1. What sorts of things are you or your relative able to discuss or have you discussed?

# ALDS hospital clinic


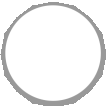

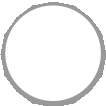

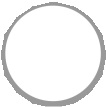

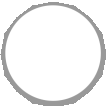

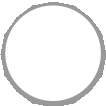


1. Do you usually have to wait long in the clinic before seeing a health professional (doctor or nurse specialist)? (Please choose only one)

Yes, but the wait is similar to other hospital clinic appointments

Yes, but the wait is shorter than my other hospital clinic appointments No

Don't know

Other (please specify)

# Specialist Respiratory Nursing Support


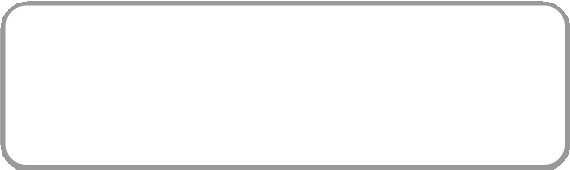

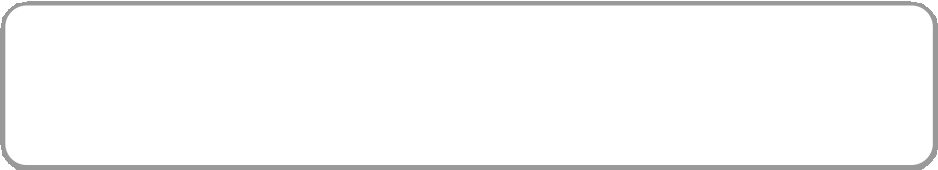

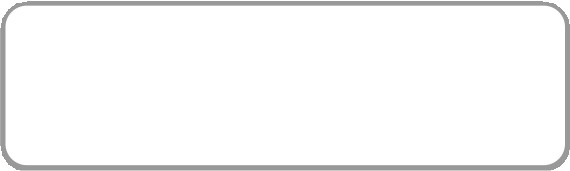

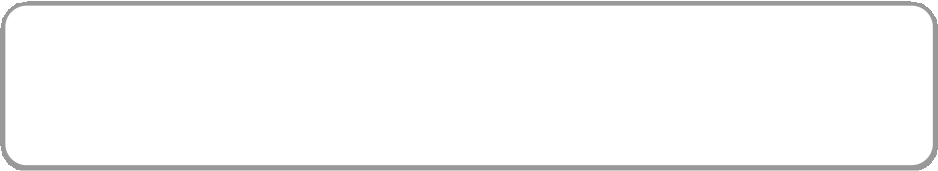

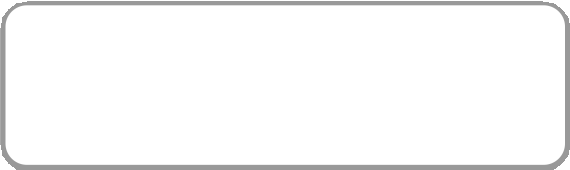

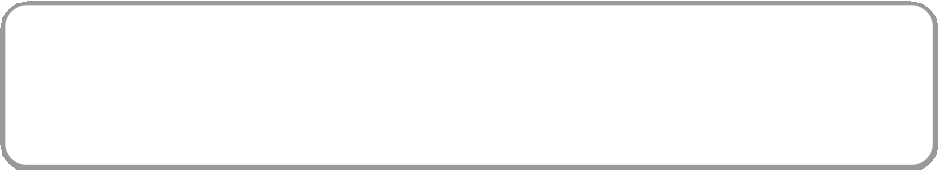

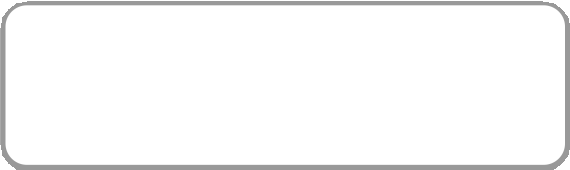

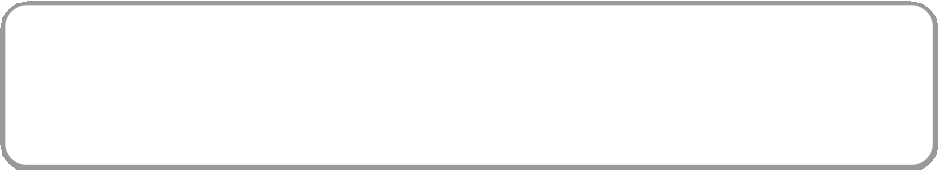

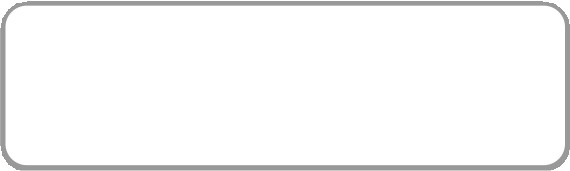

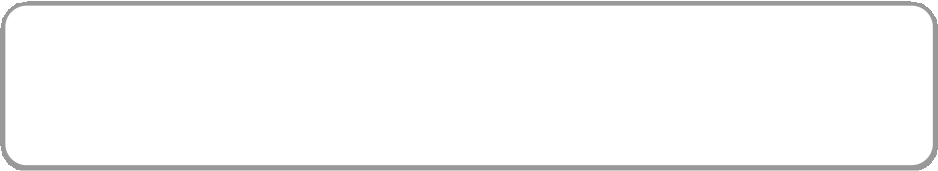


1. In addition to the hospital clinic, the Advanced Lung Disease Service offers specialist respiratory nursing support. This is provided by staff from The Royal Melbourne Hospital (RMH) and is separate to community respiratory nursing or pulmonary rehabilitation exercise programmes. Which RMH specialist respiratory nursing services have you or your relative accessed and were these helpful?

Have you used this service? Was this service helpful?

Telephone support from specialist respiratory nurse to manage appointments for clinics or tests

Telephone support from specialist respiratory nurse to manage home oxygen therapy

Telephone support from specialist respiratory nurse for advice when I am or my relative is unwell

Telephone support from specialist respiratory nurse for advice/assistance with travel, social or psychological issues

Home or nursing home visit by specialist respiratory nurse

Other specialist respiratory nursing service, please specify

# General views on the Advanced Lung Disease Service


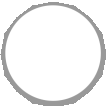

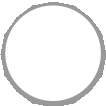

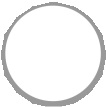

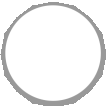

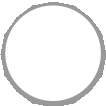

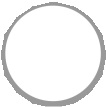

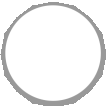

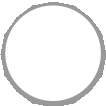

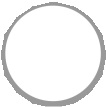

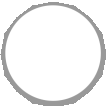

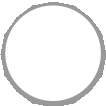

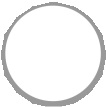

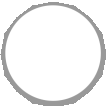

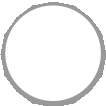

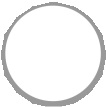

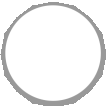


1. Does the Advanced Lung Disease Service team listen carefully to you and/or your relative? (Please choose only one)

Yes, definitely

Yes, to some extent No

Don't know

1. Do you have trust and confidence in the Advanced Lung Disease Service team? (Please choose only one)

Yes, definitely Yes, some what No

Don't know

1. Does the Advanced Lung Disease Service team treat you with respect and dignity? (Please choose only one)

Yes, definitely Yes, some what No

Don't know

1. Are you given enough time to discuss your or your relative's condition and treatment? (Please choose only one)

Yes, definitely Yes, some what No

Don't know

# General views on the Advanced Lung Disease Service


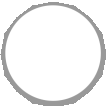

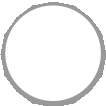

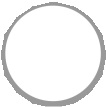

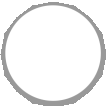

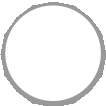

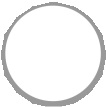

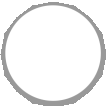

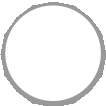

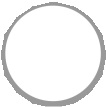


1. Are you given a chance to express your views with the Advanced Lung Disease Service team? (Please choose only one)

Yes, definitely Yes, some what No

Don't know

1. Do you have enough say in decisions about your (or your relative's) treatment and care? (Please choose only one)

Yes, definitely Yes, some what No

Don't know

Not applicable - I do not feel I should make decisions for my relative

1. The Advanced Lung Disease Service is a new and unique way of caring for people with severe lung disease. What aspects of our service are important to you or your relative? (Please select all that apply)

Long term care

Continuity of care so that I (or my relative) see(s) the same doctors and nurses Extended consultations so that I (or my relative) have more time with the clinic doctor

If I am (or my relative is) unwell I (or he/she) can be seen urgently in the Advanced Lung Disease Service clinic Afternoon appointments

RMH Respiratory nurse specialist visits the home RMH Respiratory nurse specialist telephone support

The opportunity to see a Supportive & Palliative Care doctor Other (please specify)

# Overall opinion of the Advanced Lung Disease Service


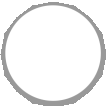

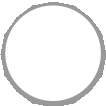

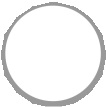

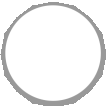

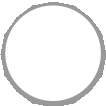

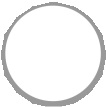

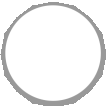

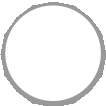

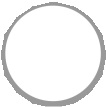

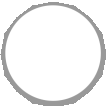


1. Would you recommend the Advanced Lung Disease Service to other people in a similar situation to you or your relative? (Please choose only one)

Yes, definitely

Yes, to some extent No

Don't know

1. Overall how would you rate the care provided by the Advanced Lung Disease Service team? (Please choose only one)

Excellent Very good Good

Fair Poor

Very poor

# How can we improve?

1. When you or your relative come to the Advanced Lung Disease Service clinic, would you like to see any other health care professionals? (Please select all that apply)

Physiotherapist Occupational therapist Psychologist

Social worker

Palliative & supportive care nurse None of the above

Other (please specify)

1. Is there any other help or support you would like to receive for yourself or for your relative from the Advanced Lung Disease Service team, or is there anything that we could do better?
2. Do you have any other comments about the Advanced Lung Disease Service?
